# Virulence factors of uropathogens and their role in host pathogen interactions

**DOI:** 10.1016/j.tcsw.2022.100075

**Published:** 2022-02-09

**Authors:** Deenadayalan Karaiyagowder Govindarajan, Kumaravel Kandaswamy

**Affiliations:** Laboratory of Molecular Biology and Genetic Engineering, Center for Excellence in Microscopy, Department of Biotechnology, Kumaraguru College of Technology, Coimbatore 641049, Tamil Nadu, India

**Keywords:** Uropathogen, Fimbriae, Surface protein, Host cell receptors, UTI

## Abstract

Gram-positive and Gram-negative bacterial pathogens are commonly found in Urinary Tract Infection (UTI), particularly infected in females like pregnant women, elder people, sexually active, or individuals prone to other risk factors for UTI. In this article, we review the expression of virulence surface proteins and their interaction with host cells for the most frequently isolated uropathogens: *Escherichia coli*, *Enterococcus faecalis, Proteus mirabilis*, *Klebsiella pneumoniae*, and *Staphylococcus saprophyticus*. In addition to the host cell interaction, surface protein regulation was also discussed in this article. The surface protein regulation serves as a key tool in differentiating the pathogen isotypes. Furthermore, it might provide insights on novel diagnostic methods to detect uropathogen that are otherwise easily overlooked due to limited culture-based assays. In essence, this review shall provide an in-depth understanding on secretion of virulence factors of various uropathogens and their role in host-pathogen interaction, this knowledge might be useful in the development of therapeutics against uropathogens.

## 1 Introduction

Urinary Tract Infection (UTI) is the most common type of Hospital Acquired Infection (HAI) that poses serious challenges in patient care (Stickler, 2008). UTI is also common among young, sexually active, and premenopausal women. Although UTI is caused by a range of pathogens, the most commonly studied UTIs are *Escherichia coli*, *Enterococcus faecalis, Proteus mirabilis*, *Klebsiella pneumoniae*, and *Staphylococcus saprophyticus* (Flores-Mireles et al., 2015). The majority of the UTIs are biofilm-associated infections, wherein pathogenic bacterial strains colonize both the tissues of the urinary tract and indwelling devices such as surgical catheters (Hatt & Rather, 2008). Catheter-Associated Urinary Tract Infections (CAUTI) is a representative type of biofilm infection where the bacterial cells colonize the surfaces of catheters and grow as biofilm communities and contain gel-like polysaccharide matrix that protects from antimicrobial compounds (Stickler, 2008). A recent study shows that a urease producing gram negative bacteria- *Proteus mirabilis* encrust catheter surfaces and blocks urine flow that leads to serious clinical complications (Pelling et al., 2019). Apart from CAUTI, studies also show that Gram-negative bacterial species were frequently isolated from 75 to 95% of non-catheter UTIs (Kahlmeter, 2003). Furthermore, some UTIs are caused by polymicrobial/multispecies interaction to host (Kline & Lewis, 2016). For instance, P*seudomonas aeruginosa* was more often associated with polymicrobial infections, whereas *Escherichia coli* (*E. coli*) was more common in monomicrobial infections (Siegman-Igra et al., 1994). Taken together, it is clear that the host surface (catheters or urinary tract) plays a major role in providing a conditioned surface on which one or more uropathogen can attach and subsequently develop as polymicrobial communities.

Besides the host surface, the cell surface of uropathogen secretes a variety of adhesive proteins that recognize specific hosts. For instance, Pili is a well-studied bacterial surface protein and has long been commonly known as a mediator of initial host-pathogen interactions and pathogenesis (Govindarajan et al., 2020, Kline et al., 2010a, Kline et al., 2010b). Pili of both gram positive and gram negative uropathogenic bacteria are decorated by multi-subunit pili proteins that are assembled via two distinct pili biogenesis pathways (Proft & Baker, 2009). One being, the chaperone/usher pathway (CUP) pili of gram-negative bacteria (eg. *Escherichia coli*), and the other being the Sortase assembled (SA) pili of gram-positive bacteria (eg. *Enterococcus Faecalis*). In addition to facilitating the host-pathogen interactions, pilin subunits are found to be crucial for virulence, colonization, tropism determination, phage transduction, DNA uptake, biofilm formation, invasion, and signalling events (Kandaswamy et al., 2013; Kline, Dodson, et al., 2010). The CUP pili (encoded by gram negative uropathogenic *E. coli*) contains various adhesins that facilitate distinct tropisms in the upper and lower urinary tract by recognizing receptors with stereochemical specificity, notably in the kidney or bladder epithelium (Wright and Hultgren, 2006). Likewise, the cell wall proteins of uropathogenic *Enterococcus faecalis* contains a variety of SA assemble pilin subunits that can facilitate colonization in kidneys by forming persistent biofilms, suggesting that the Kidney tropism was a common theme among Gram positive bacteria in the murine infection model (Horsley et al., 2013, Kau et al., 2005; Kline, Ingersoll, et al., 2010). Recent studies have reported numerous surface proteins of a variety of uropathogenic strains, however, their interactions with host tissues need a thorough review. Therefore, this article a systematic review of virulence factors of the uropathogenic strains such as *Escherichia coli*, *Enterococcus faecalis* (*E. faecalis*)*, Proteus mirabilis* (*P. mirabilis*), *Klebsiella pneumoniae* (*K. pneumoniae*), *Staphylococcus saprophyticus* (*S. saprophyticus*)*,* and *Pseudomonas aeruginosa* (*P. aeruginosa*) that are most prevalent in the majority of UTIs. In addition, this article also sheds light on the mechanistic interactions between the bacterial surface proteins and host tissues.

## 2 Uropathogen and theirinteraction to the host cells:

### 2.1 Adherence mechanism of uropathogenic *E. coli*

Uropathogenic *E. coli* (UPEC) was the leading pathogen of UTI in women, causing mortality and morbidity. Adherence of the UPEC on the host cells was the key in UTI pathogenesis (Welch et al., 2002). Two main virulence factors involved in the host cell adhesin are Type 1 fimbriae (Connell et al., 1996) and Type 2, P fimbriae (Källenius et al., 1981), Dr adhesion, S fimbriae, and F1C fimbriae.

**Fig. 1:**
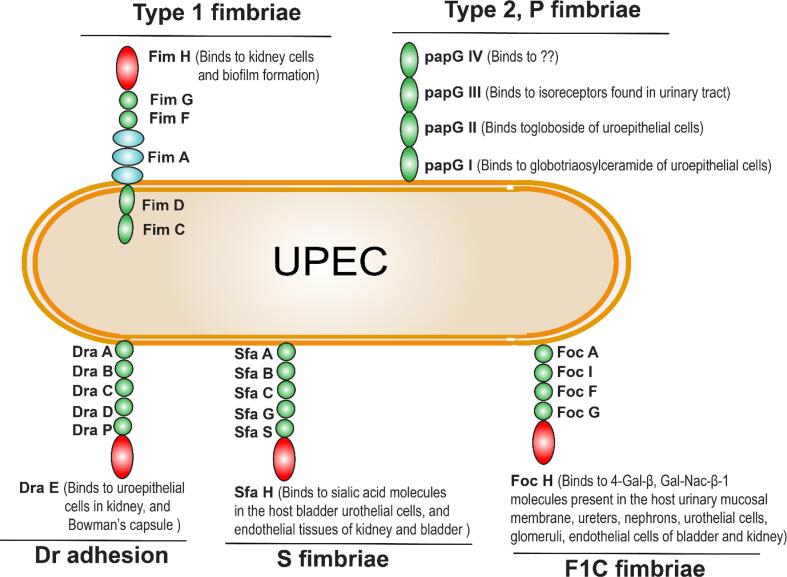
Fimbriae mediated adherence of Type 1 and Type 2, P fimbriae of UPEC. In type 1 fimbriae, the FimS, controls the (ON/OFF) of fimbrial expression and the FimB, E, A, I, C, D, F, and G are pili subunits arranged in the Type 1 fimbriae. FimH was located at the terminal end of Type 1 fimbriae binds to the kidney cells and facilitates biofilm formation during UTI. The Type 2, P fimbriae were comprised of PapGI, II, and III which binds to globotriaosylceramide or GbO_3_ of uroepithelial cells, globoside or GbO_4_, and other isoreceptors found in the urinary tract, respectively. The papGIV preferable binding receptors were unknown. Dr adhesion pili composed of DraA, B, C, D, P, and E where DraE was the pili tip binds to uroepithelial cells in kidney, and Bowman’s capsule; S fimbriae composed of SfaA, B, C, G, S, and H where SfaH was the pili tip binds to the sialic acid molecules in the host bladder urothelial cells, and endothelial tissues of kidney and bladder; The F1C fimbriae was composed of FocA, I, F, G, and H where FocH was the pili tip binds to 4-Gal-β, Gal-Nac-β-1 molecules present in the host urinary mucosal membrane, ureters, nephrons, urothelial cells, glomeruli, endothelial cells of bladder and kidney.

F pili was regulated by *fim* gene clusters. This fimbrial expression was switched ON/OFF by the invertible gene elements called *FimS* which lies in the *Fim* operon, *FimB, E, S, A, I, C, D, F, G, H* (Schwan, 2011). In the *Fim* cluster *FimB, E* mediates the orientation of *FimS* (5′- *FimS*-3′ orientation promotes the expression of fimbriae and 3′- *FimS*-5′ orientation repress the expression of fimbriae) (Bjarke Olsen and Klemm, 1994, Donato et al., 1997, Schwan et al., 1994). The FimA was the largest subunit structure and the FimH subunit was located on the tip of type 1 pili (Fig. 1), which adheres to the mannose sensitive receptor on the host cell surface (Hanson and Brinton, 1988, Krogfelt et al., 1990, Minion et al., 1986) and glycoproteins in host bladder cells (Duncan et al., 2004). *FimC* genes encodes for periplasmic chaperon protein (Orndorff and Falkow, 1984), *FimD* genes encodes usher protein (Freitag and Eisenstein, 1983). *FimF, G* controls the fimbriae length and associated with *FimH* (Schwan, 2011)*.* Another study reported that *FimH* also promotes internal bacterial community (IBC) followed by cell adhesin (Eto et al., 2007, Schembri et al., 2001, Tarchouna et al., 2013, Wright et al., 2007), aggregation on abiotic surfaces during biofilm formation (Schembri and Klemm, 2001), and formation of biofilms in rat kidneys (Melican et al., 2011).

The F pili terminal subunit, FimH binds to the mannose present in the lipopolysaccharide of neighboring bacteria forming the IBC. (Stenutz et al., 2006). The bacterial cells in the IBC were protected from antibiotics, and neutrophils. This type of colonization or biofilm formation of UPEC resides at bladder epithelium cells (Anderson et al., 2003). The outer membrane protein called antigen 43 mediates the cell–cell interaction in the IBC, F pili, and curli fibers promotes the UPEC biofilm formation by facilitating the attachment of IBC to biotic/abiotic surfaces (Guiton et al., 2012). After the maturation of IBC, it releases planktonic bacterial cells to invade other host cells and this cycle repeats (Hannan et al., 2010, Kostakioti et al., 2013). This repeated invasion and IBC formation works in favor of UPEC to survive in the host urinary track (Schwartz et al., 2011).

Unlike Type I fimbriae, Type II- P fimbriae undergo mannose-resistant adhesion on the host cell surface. The P fimbriae are encoded by *pap* genes (pyelonephritis-associated-pili Genes) such as papGI, papGII, and papGIII (Källenius et al., 1981) which facilitates the fimbriae adhesin to Galβ moieties present in human erythrocytes, host uroepithelial cells (Leffler and Edén, 1980), and globosides containing glycolipids in host kidney cells (Wright and Hultgren, 2006). Studies demonstrated that papGI adheres to the globotriaosylceramide or GbO_3_ of human uroepithelial cells, papGII adheres to the globoside or GbO_4_, (Strömberg et al., 1991) and papGIII binds with the other isoreceptors found in urinary tract of humans (Johnson et al., 2005, Johnson et al., 2001, Stapleton et al., 1998). Later studies have identified two minor papG variants such as papGI and papGIV, however, their binding mechanisms are yet to be investigated (Johnson et al., 2005, Manning et al., 2001). The interaction between the type 1 fimbriae, *Fim* genes, and P fimbriae, *pap* genes reveals that the *Fim* genes are repressed during the expression of pap genes and vice versa (Fig. 1 & Table 1). Therefore, P pili and F pili unable to undergo concurrent expression of the virulence genes (*pap* and *fim*), that limits immune stimulation during intraspecies bacterial community and these concurrent P pili and F pili expression yet to investigated in interspecies bacterial community (Holden et al., 2006). Thus, the mono expression of P pili and F pili might provide a key tool during the diagnosis and treatment of UTI.

**Table 1:** Adhesin types of uropathogen in host cell surface receptor.

Bacteria	Type of adhesin	Pili subunits / Surface Protein	Receptor surface/ host cell/abiotic surface.	Reference
UPEC	Type 1 fimbriae	FimH	Mannose receptors in the host cell surface, formation of the internal bacterial community in the host cell, and binds and formation of biofilm on biotic and abiotic surfaces like plastic and glass.	(Barras et al., 2013, Hanson and Brinton, 1988, Minion et al., 1986, Schembri et al., 2001, O'Brien et al., 2001)
	Type 2, P fimbriae	papGI	Binds to the globotriaosylceramide or GbO_3_ of human uroepithelial cells.	(Strömberg et al., 1991)
		papGII	Adheres to the globoside or GbO_4_ of uroepithelial cells.	(Strömberg et al., 1991)
		papGIII	Binds with the other isoreceptors found in the urinary tract of humans. The Type-2 fimbriae adheres to plastic and glass.	(Johnson et al., 2005, Plotkin et al., 2014)
	Dr adhesion	DraE	Binds to uroepithelial cells in kidney, and Bowman’s capsule.	(Nowicki et al., 2001)
	S fimbriae	SfaH	Binds to the sialic acid molecules in the host bladder urothelial cells, and endothelial tissues of kidney and bladder.	(Spurbeck and Mobley, 2013)
	F1C fimbriae	Foc H	Binds to 4-Gal-β, Gal-Nac-β-1 molecules present in the host urinary mucosal membrane, ureters, nephrons, urothelial cells, glomeruli, endothelial cells of bladder and kidney. It forms biofilms on abiotic surfaces like glass and plastic.	(Hancock et al., 2011, Ribić et al., 2018, Ulett et al., 2007).
*E. faecalis*	Surface protein-mediated adhesion.	Esp	Binds with the bladder cells in the mouse model and initiates biofilm formation on biotic and abiotic surface like glass, stainless steel, and plastic.	(Kristich et al., 2004, Tuomanen et al., 2001, Toledo-Arana et al., 2001)
		Ebp	Binds to the catheters and biotic surfaces which in turn induces biofilm formation on biotic and abiotic surface like glass, stainless steel, and plastic.	(Ayanto, 2021, Kline et al., 2009, Liu et al., 2020, Paganelli et al., 2012)
*P. mirabilis*	MRP fimbriae	MrpJ	Binds to mannose-resistant surfaces of bladder cells and initiates bacterial colonization. It also adheres to glass, plastic and metal surface.	(Bode et al., 2015, Jiang et al., 2020, Rocha et al., 2007b, Scavone et al., 2016)
	NAF/UCA fimbriae	UcaA	Binds with glycolipids, including asialo-GM1, asialo-GM2, and lactosylceramide of uroepithelial cells but do not contribute to UTI. It also adheres to plastic and silicon surface.	(Armbruster and Mobley, 2012, Jacobsen and Shirtliff, 2011)
	ATF	AtfA	Adhesion and formation of biofilm in the urinary tract. Also facilitates biofilm formation on glass surface.	(Scavone et al., 2016)
	PMP fimbriae	Crp	Binds to the Urinary tract. In contrast, this fimbria was regulated in diabetic patients than non-diabetic patients. It also adheres to glass surfaces.	(Armbruster et al., 2018, Filipiak et al., 2020)
	PMF	PmfA (further studies required)	Confers the bacterial colonization in bladder cells.	(Armbruster et al., 2017, Zunino et al., 2003)
*K. pneumoniae*	Type 1 fimbriae	FimA	Bladder cell invasion, formation of biofilm on bladder cells and abiotic surfaces like glass and plastic.	(Alcántar-Curiel et al., 2013, Paczosa and Mecsas, 2016)
		FimK	Binding receptors in the host cell and pili subunit function are yet to be investigated.	(Paczosa and Mecsas, 2016)
		FimH	Binds to the mannose-binding receptors in the urinary tract.	(Paczosa and Mecsas, 2016)
	Type 3 fimbriae	MrkA	Adheres and biofilm formation on abiotic surfaces like glass and plastic.	(Alcántar-Curiel et al., 2013, Allen et al., 1991)
		MrkD	Binding receptors in host cells are yet to be investigated. Studies have shown that MrkD pose-ability to bind with medical devices.	(Allen et al., 1991)
*S. saprophyticus*	Surface protein-mediated adhesion.	Aas	Binds to the fibronectin and human ureters, and bacterial colonization in rat kidneys. It also binds to abiotic plastic surface.	(Kleine et al., 2010, Wang et al., 2021)
		UafA	Binds to bladder epithelial cells.	(Kuroda et al., 2005)
		UafB	Binds to fibrinogen, fibronectin, and human bladder epithelial cells. It also binds to abiotic glass surface.	(King et al., 2011, Wang et al., 2021)
		SdrI	Binds to collagen.	(Sakinc et al., 2006)
*P. aeruginosa*	T4Pa	PilQ	Binds to the asialo GM1, 2, N-glycans, glycosphingolipid receptors present in host epithelial cells and promotes biofilm formation on biotic and abiotic surface like plastic and stainless steel.	(Craig et al., 2004, Giltner et al., 2006, Hammond et al., 2010)

The Dr adhesion was the third major notable virulence fimbriae of UPEC which causes chronic and rUTI (Jahandeh et al., 2015). Dr fimbriae comprised of six subunits such as DraA, B, C, D, P, and E which was regulated by *draA, B, C, D, P,* and *E* genes (Nowicki et al., 2001). The *draA* gene facilitates the transcriptional regulator, *draB* encodes the chaperone (DraB), *draC* encodes for the usher (DraC), *draD* mediates the invasion of fimbriae, *draP* genes contributes the mRNA cleavage mechanism (DraD), and *draE* encodes the fimbriae tip subunit, DraE which binds to the host cell surfaces (Zav'yalov et al., 2010). Like P pili, Dr fimbriae were resistant to mannose receptors but it holds several other specific receptors like α5β1-integrin, blood group antigen, type IV collagen, Type IV collagen, and Decay Accelerating Factor (DAF), carcinoembryonic antigen related cell adhesion molecules (CAECAM) (Korotkova et al., 2008, Wright and Hultgren, 2006). The aforementioned studies clearly evidences the Dr fimbriae attachment to uroepithelial cells in kidney, and Bowman’s capsule (Nowicki et al., 2001).

The S fimbriae was in UPEC was structurally resembles the P pili, F pili, and F1C (Chahales and Thanassi, 2015). The S fimbriae was encoded by *sfaC, B, A, D, E, F, G, S,* and *H* (Zaw and Lin, 2017). The genes *sfaC, B* encodes the regulatory proteins (SfaC, B), *sfaC* encodes the major fimbriae subunit (SfaA), *sfaE, F* encodes the usher and chaperon subunits, *sfaG, S* encodes the minor subunits (SfaG, S), and *sfaH* encodes the fimbriae tip subunit (SfaH) (Balsalobre, Morschhäuser, Jass, Hacker, & Uhlin, 2003; Werneburg and Thanassi, 2018). Interestingly, the regulatory proteins SfaC, B mediates the presence/absence of S fimbriae in UPEC whereas these regulatory proteins are suppressed by the factors like environmental temperature and small molecules like glucose (Balsalobre et al., 2003). The fimbriae tip protein SfaH adheres to the sialic acid molecules in the host bladder urothelial cells, and endothelial tissues of kidney and bladder (Spurbeck and Mobley, 2013).

The F1C fimbriae was one of the fimbriae found in the UPEC. The F1C fimbriae was encoded of *focA, I, C, D, F, G,* and *H* (Zaw and Lin, 2017). whereas the *focA* encodes the major pili subunit FocA, *focI* encode the fimbriae regulatory protein subunit FocI, *focC, D* encodes the chaperone and usher proteins, *focF, G* encodes the minor subunit FocF and FocG, *focH* encodes the fimbriae tip subunit FocH (Werneburg and Thanassi, 2018). The fimbriae tip FocH adheres to 4-Gal-β, Gal-Nac-β-1 molecules present in the host urinary mucosal membrane, ureters, nephrons, urothelial cells, glomeruli, endothelial cells of bladder and kidney (Ribić et al., 2018).

The F1C genes were similar to the F pili operons *fimA, I, C, D, F, G, I,* and *H* (Behzadi, 2018). In contrast, the F pili and P pili genes are downregulated during the expression of F1C fimbriae, i.e., the F1C fimbriae was found in the absence of F pili and P pili, vice versa. Also, they target different binding receptors (Chahales and Thanassi, 2015).

From the aforementioned, it is clear that UPEC can able to express either *pap* genes or *fim* genes or *foc* genes during fimbriae synthesis, all together cannot be expressed. These fimbriae genes produce the fimbriae/pili/virulence proteins based on their peripheral conditions, whereas upregulation of one gene in turn downregulates the other two genes. It provides the possible diagnostic method to provide competitive inhibitors to inhibit the regulatory pili proteins and stops bacterial adhesin to the host. The co-expression of *sfa* gene and *dra* gene during their fimbriae synthesis was not concrete.

### 2.2 Adherence mechanisms of uropathogenic *E. faecalis*

*E. faecalis*, a Gram-positive diplococcus bacterium that occurs in short chains which causes CAUTI and HAI (Hidron et al., 2008, Maki and Tambyah, 2001, Richards et al., 1999). *E. faecalis* infection was also studied in diabetic and non-diabetic women shown a controversial finding, diabetic women were more prone to UTI (Boyko et al., 2005, Nicolle et al., 1996) and vice versa (Bonadio et al., 2006). This complicated finding reveals that diabetes was a risk factor for accenting UTI in rats (Raffel et al., 1981) and murine models (Rosen et al., 2008a, Rosen et al., 2008b). Physiologically *E. faecalis* does not contains flagella or pili but adherence to the host cell by their surface proteins such as Esp (Enterococcal Surface Proteins) (Shankar et al., 1999) and Ebp (Endocarditis and biofilm-associated pilus).

The Esp was the larger surface protein with repetitive domains which is homologous to the C alpha determinants and Rib determinants in streptococci which provides bacterial persistence to antibiotics (Larsson et al., 1996, Li et al., 1997, Madoff et al., 1996, Tuomanen et al., 1999). Esp proteins facilitate the *E. faecalis* adherence to fibrinogen and collagen ligands presents in bladder cells in the mouse model and also facilitate the biofilm formation (Tuomanen et al., 2001, Tendolkar et al., 2004).

The Ebp protein was composed of EbpA, B, and C, shows binding affinity to the host cells and early biofilm formation. Physiologically, Ebp was assembled on the cell wall by the membrane anchoring proteins called SortaseA, C (SrtA and SrtC) (Kline et al., 2009, Nallapareddy et al., 2006, Nielsen et al., 2013, Schlüter et al., 2009). *In vitro* studies have shown that SrtA and SrtC-Ebp regulates the initial biofilm formation during UTI (Guiton et al., 2009, Nallapareddy et al., 2006). In addition, *in vivo* studies shows that SrtA and Ebp regulate the biofilm formation during CAUTI (Guiton et al., 2010, Nielsen et al., 2012). In addition to sortase proteins, several pilus components also promote the Ebp in adherence and early biofilm formation are initiated with Agg (aggregation substances), Ace (adhesin to collagen). Ace proteins mainly involve in the formation of biofilm (Roh et al., 2015) and bacterial colonization in the rat UTI model (Lebreton et al., 2009). Whereas, Agg binds to the renal epithelial cells (Rozdzinski et al., 2001) and lipoteichoic acid (LTA) in neighboring *E. faecalis* cells (Waters et al., 2004). In contrast, the following showed that secretion of LTS inhibits the bacterial adherence to the host (Wobser et al., 2014). Therefore, the role of LTA during the adhesin protein expressions remains unclear.

**Fig. 2:**
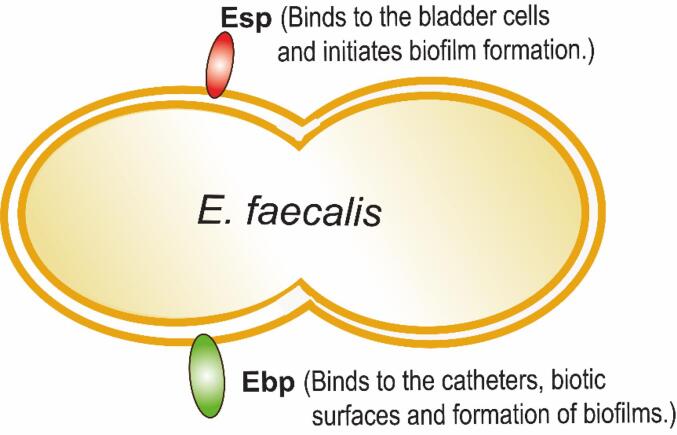
Surface protein, Esp and Ebp mediated adherence of *E. faecalis*. In *E. faecalis* UTI, surface proteins mediated the bacterial cell adherence to the host cell surfaces i.e., Esp proteins bind to the bladder cells, Ebp proteins binds to the biotic and abiotic surfaces like catheters, and both surface proteins pose-ability in the formation of biofilm.

All the aforementioned surface proteins promote ascending UTI in a mouse model in the absence of catheter (Kemp et al., 2007, Sillanpää et al., 2013, Singh et al., 2007). Taken together, it is clear that Ebp plays a major role in the biofilm formation on the biotic and abiotic surface during UTI. (Fig. 2 & Table 1). Notably the Ebp pili confers infection in the absence of medical catheters are resourced by bacterial communities and dispersion of biofilm. These biofilm formation was regulated by the intracellular cell signaling which was termed as quorum sensing by pheromone, these peptides coordinate the gene expression and biofilm formation in enterococcal species (Cook and Federle, 2014).

### 2.3 Adherence mechanisms of uropathogenic *P. mirabilis*

**Fig. 3:**
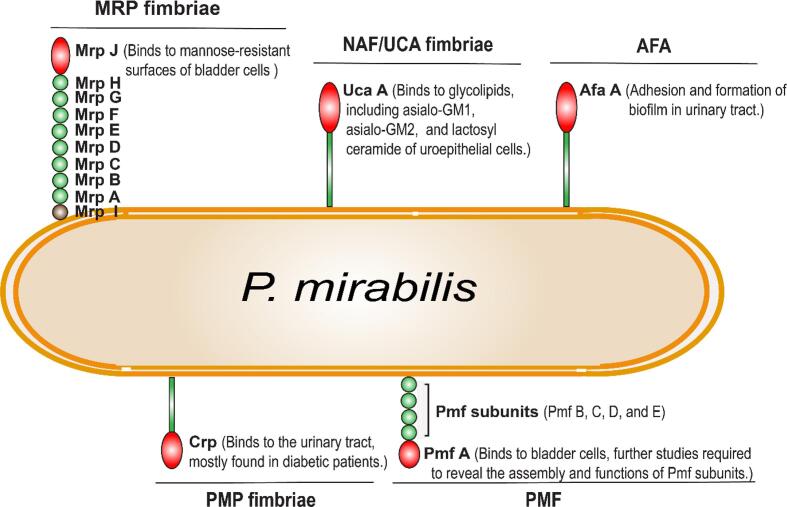
Fimbriae mediated adherence of *P. mirabilis*. *P. mirabilis* contains several pili around the cell surface which are categorized based on their adherence mechanism, MRP fimbriae was expressed during the ON phase of *MrpI* codons and vice versa. The MrpH subunit located at the terminal end of MRP fimbriae binds to the mannose-resistant surfaces of bladder cells and initiates bacterial colonization. In NAF/UCA fimbriae, The UcaA subunit binds with glycolipids, including asialo-GM1, asialo-GM2, and lactosyl ceramide of uroepithelial cells but does not contribute to UTI. In ATF, AtfA pili subunits pose-ability of bacterial cell attachment and biofilm formation in the urinary tract. In PMF, Pmf pili subunits assembly and their functions were poorly studied, PmfA subunits were recognized for binding to the host bladder cells and several studies were required to reveal their specific mechanism.

*P. mirabilis* is known for the complicated UTI which adheres to the host cells unlike other bacterial cell’s morphology, *P. mirabilis* express several fimbriae such as mannose-resistant Proteus fimbriae (MRP fimbriae), ambient temperature fimbriae, uroepithelial cell adhesion, P*. mirabilis* P-like fimbriae, and *P. mirabilis* fimbriae (Rocha et al., 2007a, Rocha et al., 2007b). The mannose-resistant Proteus fimbriae (MRP fimbriae) facilitates homo aggregation of *P. mirabilis* and initial biofilm formation (Jansen et al., 2004). The *mrp* operons (*mrpA, B, C, D, E, F, G, and H*) control the synthesis of mannose-resistant *Proteus* fimbriae. The *mrpI* as operon switch possesses a similar function of *Fim* genes in UPEC. The *mrpI* ON Phase facilitates the fimbrial expression and vice versa. During the fimbrial expression, mrpJ, pilus subunit presents in the terminal end binds to the mannose-resistant surfaces of bladder cells and initiates the bacterial colonization (Fig. 3 & Table 1). The *mrpI* invertible operon on OFF phase results in absence of fimbrial expression (Bode et al., 2015, Li et al., 2002, Li et al., 2001). The *mrpI* switching was influenced by integration host factor, histidine-like nucleoid structured protein, and DNA methylation or heat unstable nucleoid protein (X. Li et al., 2002).

The uroepithelial cell adhesion (UCA) fimbriae or Non-agglutinating fimbriae (NAF) are facilitated by the *ucaA* gene (Cook et al., 1995). The UCA fimbriae bind to glycolipids, including asialo-GM1, asialo-GM2, and lactosyl ceramide of uroepithelial cells but do not contribute to UTI (Fig. 3 & Table 1) (Armbruster and Mobley, 2012, Lee et al., 2000).

Early studies states that Ambient Temperature Fimbriae (ATF) are expressed under the ambient temperature (25 °C) which was inhibited by host body temperature (37 °C), and ATF doesn’t take place in bacterial colonization on the urinary tract (Zunino et al., 2000). Recent studies revealed that ATF in *P. mirabilis* isotypes plays a major role in the adhesion and formation of biofilm in the urinary tract (Fig. 3 & Table 1) (Scavone et al., 2016). The ATF fimbriae are regulated by *atfA* gene (Kuan et al., 2014).

The *P. mirabilis* P-like (PMP) fimbriae are regulated by *Crp* genes, the Crp associated PMP fimbriae facilitates the *P. mirabilis* colonization in host kidney epithelial cells of the diabetic host than the nondiabetic host (Fig. 3 & Table 1). It was found that the glucose level present in diabetic and nondiabetic host cells mediates the *Crp* expression and loss of *Crp* genes, respectively. Also, the glucose-mediated *Crp* expression upregulates the synthesis of PMP fimbriae (Armbruster et al., 2018).

The *P. mirabilis* fimbriae (PMF) is regulated by *pmfACDEF* operon (Pearson et al., 2011). The gene expression studies revealed that *pmfA* mutants do not infect the host kidney cells compared to wild-type (Zunino et al., 2003). In another study, it was shown that PMF confers the bacterial colonization in the bladder but the exact contribution of fimbrial expression remains unknown (Zunino et al., 2003) (Fig. 3 & Table 1).

### 2.3 Adherence mechanisms of uropathogenic *K. pneumoniae*

**Fig. 4:**
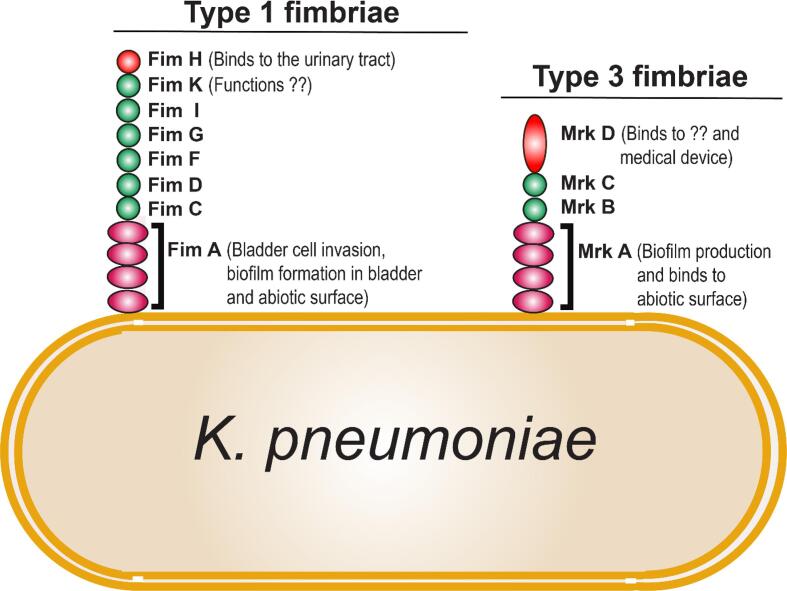
Fimbriae mediated adherence of Type 1 and Type 3 fimbriae of *K. pneumoniae*. In type 1 fimbriae, the FimA, a huge pili subunit facilitates the bladder cell invasion, and biofilm formation in bladder cells and abiotic surfaces, FimH subunit present at the tip of Type 1 fimbriae attach to the mannose receptors in the host cell surface. Type 3 fimbriae MrkA, a huge pili subunit facilitates biofilm production and also aggregates to the abiotic surfaces, MrkD subunits present at the tip of Type 3 fimbriae attached to the medical devices.

*K. pneumoniae,* was a widely known opportunistic pathogen that causes bacteremia, septicemia, and nosocomial pneumonia, and also an opportunistic pathogen for UTI in women (Ronald, 2002). Previous reviews have shown that *K. pneumoniae* was the second species after UPEC which has been found in women with recurrent UTI (Stamm et al., 1991). *K. pneumoniae* facilitates two types of fimbrial adhesin, type 1 fimbriae and type 3 fimbriae for the bacterial colonization in the host cells (Podschun and Ullmann, 1998). Type 1 fimbriae are adherent to the mannose-containing structures of the host cells. The type 1 fimbriae were regulated by the invertible gene element called *fim* switch (Klemm and Schembri, 2000), FimH pili subunit present at the terminal end performs fimbrial adhesion to mast cells and promotes mannose sensitive binding. FimA subunit binds to the bladder cell and promotes cellular invasion and biofilm formation on bladder cells and other abiotic surfaces (Fig. 4 & Table 1). The other minor subunits such as FimC, D, F, and G are functionally similar to UPEC Fim subunits. (Paczosa and Mecsas, 2016). In addition, FimK, an additional gene cluster present in *K. pneumoniae* which was absent in UPEC but exact function is yet to be investigated.

Type 3 fimbriae synthesis is mediated by the expression of *mrkA, B, C, and D* gene clusters (Tarkkanen et al., 1998). MrkA is the larger subunit and the minor subunit/adhesin MrkD is located at the tip of the of pili (Allen et al., 1991). Similar to Type 1 fimbriae, Type 3 fimbriae operons were present and synthesized by most *K. pneumoniae* isolates but the Type 3 fimbriae were insensitive to mannose receptors. The Type 3 fimbriae-specific host cell surface receptors are yet to be investigated.

The *Fim* switches facilitate the Type 1 fimbriae expression on the UTI but not on the lungs or gastrointestinal tracts in the mouse model (Struve et al., 2008, Struve et al., 2009). *In*- *vivo* studies demonstrated that Type 1 fimbriae enable the bacteria to attach to the bladder cells and promotes the formation of biofilm during the UTI (Rosen, Pinkner, et al., 2008). Given the aforementioned findings, Struve et al. revealed that mutants lacking *Fim* genes adhere only to the lung, spleen, and liver cells (Struve et al., 2009). Likewise, The Type 3 fimbriae do not confer *K. pneumoniae* colonization in the Gastro-Intestinal tract and lungs. Similarly, Type 3 fimbrial expression of *K. pneumoniae* was found only in *in vitro* studies and the Type 3 fimbriae was not expressed in *in vivo* mouse model, this concludes that Type 3 fimbriae do not contribute to the UTI (Würker et al., 1990). In addition, Type 3 fimbriae expression was observed during the biofilm aggregation on the abiotic surfaces like catheters (Fig. 4 & Table 1) (Jagnow and Clegg, 2003, Stahlhut et al., 2012). In general, biofilms formed on the catheters are considered to be a reservoir for CAUTI. The bacterial cells within the extracellular matrix of biofilms are often resistant to antibiotics and chemicals where the planktonic bacterial cells are susceptible (Davies, 2003). These biofilms pose-ability to transmit the *K. pneumoniae* cells on the catheter entry site of the patients which directly results in UTI.

### 2.4 Adherence mechanisms of uropathogenic *S. saprophyticus*

**Fig. 5:**
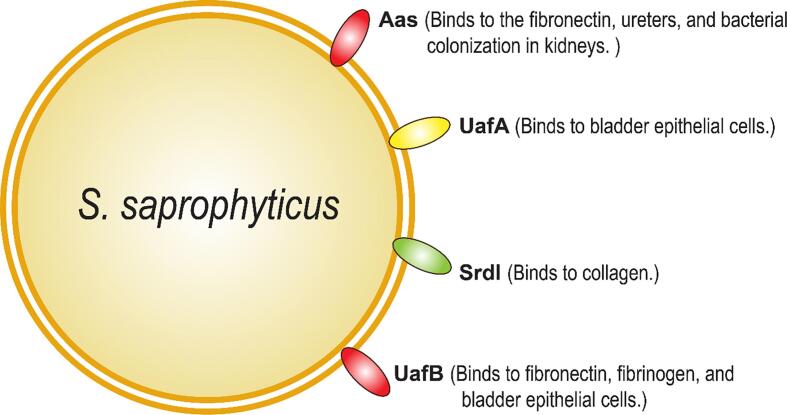
Surface protein-mediated adherence of *S. saprophyticus*. In *S. saprophyticus* UTI, surface proteins facilitate the bacterial adherence to the host cell surfaces i.e., p Aas proteins bind to human ureters and fibronectin, and bacterial colonization in rat kidneys. The UafA proteins bind to bladder epithelial cells. UafB binds to fibrinogen, fibronectin, and human bladder epithelial cells and the SdrI binds to the collagen during the UTI.

*S. saprophyticus* is a Gram-positive bacterium that leads to uncomplicated UTI as cystitis in sexually active women (Ehlers and Merrill, 2018). Although *S. saprophyticus* shares many clinical features like transmission through sexual intercourse leads to UTI caused by *E. coli*, but the *S. saprophyticus* colonization was predominantly found in female and rarely found in male (Colodner et al., 2006). However, *S. saprophyticus* differs in pathogenesis as their virulence proteins are very distinct (Raz et al., 2005). The surface assembled virulence factors include several adhesins and cell wall-anchored (via sortase enzymes) virulence proteins such as Aas, UafA, UafB, SdrI, and SssF (Kline and Lewis, 2016). Autolysin/adhesin of *S. saprophyticus* (AasAas) is a cell-wall associated protein that has autolytic and adhesive properties. Aas is conserved among all *S. saprophyticus* strains and has an affinity towards fibronectin and human ureters in vitro, and has been found to colonize rat kidneys *in vivo* (Fig. 5 & Table 1) (Hell et al., 1998, Kleine et al., 2010).

Uro-adherence factor A (Uaf A) is a hemagglutinin that has an affinity to bladder epithelial cells *S. saprophyticus* strains examined to date under in-vitro conditions (Kuroda et al., 2005). However, Plasmid-encoded UafB, is a serine-rich glycoprotein, found only in ∼5% of strains examined, and binds to fibrinogen, fibronectin, and human bladder epithelial cells but does not promote bladder colonization in a murine UTI model (King et al., 2011). SdrI is another cell wall-associated serine-aspartate-rich protein that binds to collagen, associated with bacterial surface hydrophobicity, found only in a minority of *S. saprophyticus* strains, and plays a major role in acute UTI and persistent kidney infections (Fig. 5 & Table 1) (King et al., 2011, Kline et al., 2010b, Kline et al., 2009, Sakinc et al., 2006). Both Ssp and SdrI proteins are also required for the bacterial persistence in kidney and bladder. Furthermore, *S. saprophyticus* also encodes for urease that is responsible for persistent bacterial colonization in bladder, kidneys, and for dissemination to the spleen in a rat model of UTI (Gatermann et al., 1989). Furthermore, the urease enzyme catalyzes the urea into ammonia and carbon dioxide which in turn forms carbonate precipitation (stone formation) and causes severe damage to the host kidney tissue, uroepithelium and formation of abscesses (Gatermann et al., 1989).

### 2.5 Adherence mechanisms of uropathogenic *P. aeruginosa*

The uropathogenic *P. aeruginosa* was an opportunistic pathogen causing CAUTI in humans which was a third notable strains of UTI in hospital settings after *E. coli* and *E. faecalis* (Cole et al., 2014). The *P. aeruginosa* causes nosocomial UTI via catheter associated infection in humans, whereas the catheter associated bacteriuria extend the patients stay in the hospital (Warren, 2001). Initially, *P. aeruginosa* biofilms adheres on the catheter surfaces and reaches the human urinary tract and initiates their pathogenicity. Whereas the *P. aeruginosa* biofilm matrix composed of extracellular polysaccharides, alginate, eDNA, and proteinaceous components (Ryder et al., 2007). The extracellular polysaccharides like PEL and PSL by *pel* and *psl* genes were found to be a key role formation of biofilm (Friedman and Kolter, 2004, Jackson et al., 2004). In the early reviews, the PEL and PSL’s structural role in the biofilm formation remains unknown (Mittal et al., 2009). In the following studies reveal that pel was a cationic exopolysaccharide, psl polysaccharide does not contain any charge, and alginate was an anionic polysaccharide. Form the aforementioned it was clear that, *P. aeruginosa* can able to synthesize differently charged polysaccharide at their physiological pH provides the stability to their biofilm matrix. Furthermore, the positively charged Pel polysaccharide can directly interact with the host cell surfaces containing mucin and hyaluronan.

Whereas the antibiotic treatment on the catheters and humans eliminates the planktonic *P. aeruginosa* but the bacteria inside the biofilm matrix remains unaffected (Stewart and Costerton, 2001). In the post antibiotic treatment, the bacteria inside the biofilm matrix provides a set of *P. aeruginosa* planktonic bacteria which causes the recurrent infection (Srinivasan et al., 2021). The planktonic *P. aeruginosa* was a gram negative monoflagellated rod-shaped bacteria, and use their flagella and the other cell surface components to interact with the host cell surface. The adherence factors are, flagella interact to mucin (both cell-associated mucin and secreted mucin), Toll-like receptors 5, asialo GM1, and heparin sulfate proteoglycans of host epithelial cells and initiates bacterial invasion (Hayashi et al., 2001, Bucior et al., 2012).

**Fig. 6:**
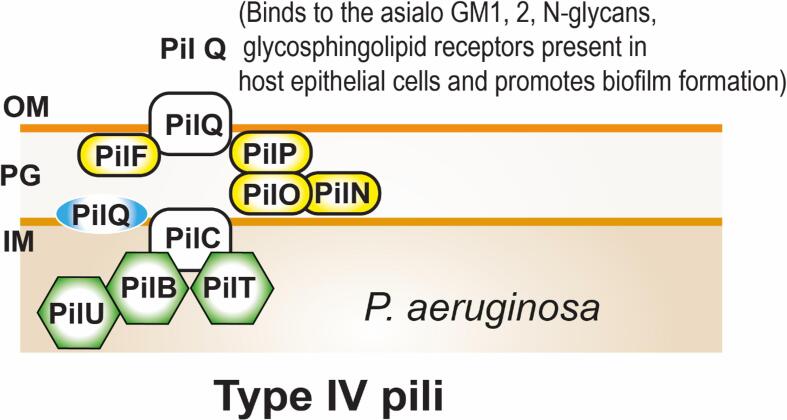
Surface protein-mediated adherence of *P. aeruginosa*. In *P. aeruginosa* UTI, surface proteins facilitate the bacterial adherence to the host cell surfaces i.e., PilQ, secretory proteins bind to the asialo GM1, 2, N-glycans, glycosphingolipid receptors present in host epithelial cells and promotes biofilm formation. (OM- Outer membrane, IM- Inner mebrane, and PG- Peptidoglycan).

In addition to flagella, type IV pili (T4P) present in *P. aeruginosa* which adheres to the host epithelial cells, and initiates the biofilm formation (Winstanley et al., 2016). Type IV pili was the major factor in host-bacterial adherence (Bucior et al., 2012). The Type IV pili are divided into three subtypes such as T4Pa, T4Pb, and T4Pb-Tad. The T4Pa comprised of PilA, B, C, F, M, N, P, T, and U are pili subunits where PilQ are the secretory protein which involves in the host cell interaction (Fig. 6 & Table 1). In the similar fashion PilS2, Q2, T2, R2, O2, L2, and P2 are the T4Pb subunits and PilN2 was a secretory protein (Carter et al., 2010, de Bentzmann et al., 2006). The T4b-Tad contains TadA, B, C, D, G, Z, and Flp are pili subunits and the RcpA was a secretory protein (Cole et al., 2014). The T4Pa adheres to the asialo GM1, 2, N-glycans, glycosphingolipid receptors present in host epithelial cells (Craig et al., 2004). The T4Pb and T4Pb-Tad were not associated with motility and its receptor binding sites yet to be investigated. Also, T4P promotes the surface attachment of *P. aeruginosa* cells, microcolony formation, and biofilm formation (O'Toole and Kolter, 1998).

In addition to pili and flagella, *P. aeruginosa* contains secretion systems such as type I, II, III, V, and VI secretion system. These secretary systems involved the transportation of virulence factors extracellular environment but does not promotes bacterial adhesin (Wu et al., 2015).

## 3 Multispecies biofilm reservoir for UTI

In natural settings, most microorganisms were present in complex communities called polymicrobial biofilms. These polymicrobial biofilms are also found in the medical catheters which recods about 86% of CAUTI was polymicrobial infections (Percival et al., 2015). Galván et al. shown the uropathogenic interaction between two species such as *K. pneumoniae-E. coli*, *E. coli-E. faecalis, K. pneumoniae-P. mirabilis,* and *E. faecalis-K. pneumoniae*. Firstly, *K. pneumoniae-E. coli* cultures shown reduced *E. coli* viable cells and reduced *E. coli* biofilm dispersal when compared to single species cultures. On the other hand, *K. pneumoniae* cells dispersal from the dual species biofilms was similar to single species biofilms. The similar fashion was found in the planktonic cells (Galvan et al., 2016). In the following study, Galván et al. revealed that *K. pneumoniae* can able to produce more siderophore and also survive under iron limited conditions when compared to *E. coli* in mixed species biofilms (Juarez and Galván, 2018). Secondly, viable *K. pneumoniae* cells were reduced when cultivated together with *P. mirabilis* in both biofilm and planktonic samples. Thirdly, *K. pneumoniae-E. faecalis* biofilm are found to disperse increased viable cells from mixed and single species biofilm model. Also, planktonic *K. pneumoniae-E. faecalis* bacterial cells also posed positive growth effect. This cooperative growth behaviour could be modified gene expression in interbacterial communication during mixed biofilms (Lazazzera, 2005). Fourthly, the bacterial interaction between *E. coli* and *E. faecalis* shown positive effect in both biofilm formation and planktonic cultures (Galván et al., 2016). In addition, another study revealed that *E. coli* gets benefitted by L-ornithine secreted by *E. faecalis*, also it enables *E. coli* to survive under iron limited condition (Keogh et al., 2016). Fifthly, *P. aeruginosa-K. pneumoniae-P. protegenes* shown the overproduction of alginate and Psl which provides antibiotic tolerance to the *P. aeruginosa* biofilms (Periasamy et al., 2015). On the other hand, Psl provides antibiotic tolerance to *Staphylococcus aureus-E. coli* (Billings et al., 2013)*.* Finally, from the aforementioned it is clear that probabilities of polymicrobial infection from mixed biofilms. The molecular level interaction between interspecies and role of secreted compounds effect on mixed bacterial species remains unknown. In addition, the other couple bacterial interactions such as *P. mirabilis*-*E. coli, E. faecalis; P. aeruginosa-* uropathogens (*E. coli*, *E. faecalis, P. mirabilis*, and *S. saprophyticus*) and *S. saprophyticus*- uropathogens (*E. coli*, *E. faecalis, P. mirabilis*, *K. pneumoniae,* and *P. aeruginosa*) along with the expression of adhesin and virulence proteins yet to be investigated. The future findings for the aforementioned research gap might provide us the possible insides in controlling polymicrobial infections.

## 4 Conclusion and perspectives

In this review, we discussed the mono expression of UPEC fimbriae i.e., Type 1 or Type 2 fimbriae as a virulent fimbrial protein, the cross-talk between the Type 1 fimbriae and Type 2 fimbriae revealed that either one of the pili type can express during the pathogenesis but not both (Holden et al., 2006). Based on these Pili functions, emerging studies on phytochemicals and plant-peptides showed inhibition to UPEC along with *in silico* insights in predicting the protein-drug interaction (Jaiswal et al., 2019). However, one has to conduct studies on fimbrial expression in the presence of phytochemicals and antimicrobial compounds from plant sources to better devise a strategy for the prevention of UTI. Similarly, phytochemical screening against the uropathogen for other strains, *E. faecalis* (D’Sousa’Costa et al., 2015), *P. mirabilis* (Pidugu and Arun, 2012), *K. pneumoniae* (Dahiya and Purkayastha, 2012), and *S. saprophyticus* (Kumar et al., 2011) shows *in-vitro* inhibition of bacterial strains but their efficacy in humans need further investigation. In addition, further studies on the fimbriae and surface protein expression under the phytochemical stress might provide novel compounds for the prevention of UTI.

UTIs are the most common type of bacterial infection that results in significant healthcare problems (Stamm and Norrby, 2001). The uropathogen that encode wide range of virulence factors that are predominantly localized on cell surfaces, suggest that the treatment of UTI should be more focussed on virulence determinants the essential for initial attachment (Eg. FimH of UPEC). **A**dministration of antibiotic to treat UTI was proven to be ineffective due to the spread of drug resistant strain types. Therefore, chemical compounds such as mannosides and pilicides could be a promising alternative as they are proven to be effective in neutralizing pathogenic bacteria and prevent disease in animal models (Flores-Mireles et al., 2015, Greene et al., 2014). In another study it was shown that consumption of cranberry juice could mitigate the severity of UTI and does not significantly alter the gut microbiota (L. E. Nicolle, 2016, Straub et al., 2021). Several chemical compounds such as forskolin (Bishop et al., 2007), anti-FimH antibodies (Thankavel et al., 1997), small molecules (Bouckaert et al., 2005), and N-butyldeoxynojirimycin (Svensson et al., 2003) considered as potent drugs to treat UTI in animal models are currently in preclinical stages of development. It is important to note that the aforementioned compounds are not tested in humans but protamine sulfate which inhibits the bacterial invasion in the mouse model was tested in humans and reported to cause discomfort during the treatment (Lilly and Parsons, 1990). Given these findings, it is important to note that more translational research is needed to devise a strategy for UTI treatments that do not alter the normal microflora.

Finally, substantial efforts are needed in conducting future clinical trials that are essential in translating the aforementioned novel anti-virulence therapies into new treatments that can reduce healthcare burden associated with UTIs.

## CRediT authorship contribution statement

**Deenadayalan Karaiyagowder Govindarajan:** Conceptualization, Data curation, Writing – original draft, Visualization. **Kumaravel Kandaswamy:** Investigation, Supervision, Writing – review & editing.

## Declaration of Competing Interest

The authors declare that they have no known competing financial interests or personal relationships that could have appeared to influence the work reported in this paper.
